# Supplementary material for: Evolution of (p)ppGpp-HPRT regulation through diversification of an allosteric oligomeric interaction
Source: eLife. 2019 Sep 25;8:e47534. doi: 10.7554/eLife.47534 (PMC6783271; doi:10.7554/eLife.47534)
Supplement: Table 2—source data 2. [file elife-47534-table2-data2.pdf]

Preliminary Full wwPDB X-ray Structure Validation Report (i)

Sep 18, 2017 – 06:25 PM EDT

This is a Preliminary Full wwPDB X-ray Structure Validation Report.

This report is produced by the standalone wwPDB validation server.
The structure in question has not been deposited to the wwPDB.
This report should not be submitted to journals.

We welcome your comments at *validation@mail.wwpdb.org*
A user guide is available at
http://wwpdb.org/validation/2016/XrayValidationReportHelp
with specific help available everywhere you see the (i) symbol.

PRELIMINARY VALIDATION REPORT

The following versions of software and data (see references (i)) were used in the production of this report:

MolProbity	:	4.02b-467
Mogul	:	1.7.2 (RC1), CSD as538be (2017)
Xtriage (Phenix)	:	1.9-1692
EDS	:	rb-20029824
Percentile statistics	:	20161228.v01 (using entries in the PDB archive December 28th 2016)
Refmac	:	5.8.0135
CCP4	:	6.5.0
Ideal geometry (proteins)	:	Engh & Huber (2001)
Ideal geometry (DNA, RNA)	:	Parkinson et al. (1996)
Validation Pipeline (wwPDB-VP)	:	rb-20029824

1 Overall quality at a glance (i)

The following experimental techniques were used to determine the structure:
X-RAY DIFFRACTION

The reported resolution of this entry is 1.64 Å.

Percentile scores (ranging between 0-100) for global validation metrics of the entry are shown in the following graphic. The table shows the number of entries on which the scores are based.

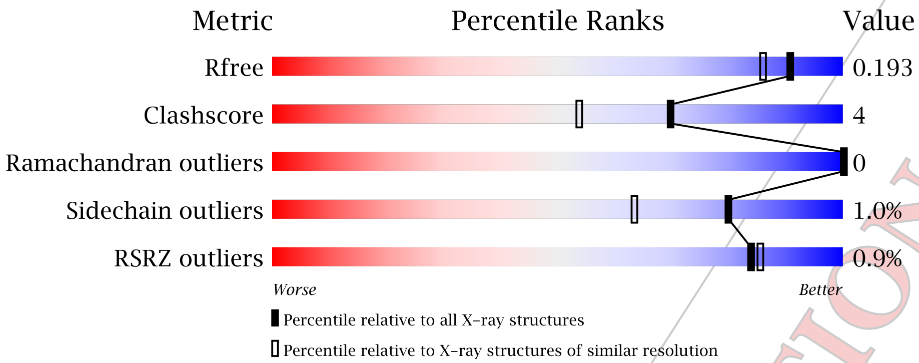

Metric	Whole archive (#Entries)	Similar resolution (#Entries, resolution range(Å))
R_{free}	100719	2211 (1.66-1.62)
Clashscore	112137	2356 (1.66-1.62)
Ramachandran outliers	110173	2315 (1.66-1.62)
Sidechain outliers	110143	2315 (1.66-1.62)
RSRZ outliers	101464	2219 (1.66-1.62)

The table below summarises the geometric issues observed across the polymeric chains and their fit to the electron density. The red, orange, yellow and green segments on the lower bar indicate the fraction of residues that contain outliers for >=3, 2, 1 and 0 types of geometric quality criteria. A grey segment represents the fraction of residues that are not modelled. The numeric value for each fraction is indicated below the corresponding segment, with a dot representing fractions <=5% The upper red bar (where present) indicates the fraction of residues that have poor fit to the electron density. The numeric value is given above the bar.

Mol	Chain	Length	Quality of chain
1	A	173	.% / 94% / 6%
1	B	173	.% / 85% / 14% / .

The following table lists non-polymeric compounds, carbohydrate monomers and non-standard residues in protein, DNA, RNA chains that are outliers for geometric or electron-density-fit criteria:

PRELIMINARY VALIDATION REPORT

Mol	Type	Chain	Res	Chirality	Geometry	Clashes	Electron density
5	PEG	G	4	-	-	-	X
6	ACT	H	1	-	-	-	X
6	ACT	H	3	-	-	-	X
7	EDO	I	10	-	-	-	X
7	EDO	I	11	-	-	-	X
7	EDO	I	3	-	-	-	X
7	EDO	I	6	-	-	-	X
7	EDO	I	8	-	-	-	X

PRELIMINARY VALIDATION REPORT

2 Entry composition (i)

There are 8 unique types of molecules in this entry. The entry contains 6487 atoms, of which 3118 are hydrogens and 0 are deuteriums.

In the tables below, the ZeroOcc column contains the number of atoms modelled with zero occupancy, the AltConf column contains the number of residues with at least one atom in alternate conformation and the Trace column contains the number of residues modelled with at most 2 atoms.

- Molecule 1 is a protein.

Mol	Chain	Residues	Atoms						ZeroOcc	AltConf	Trace
1	A	173	Total 2837	C 918	H 1431	N 215	O 266	S 7	0	7	0
1	B	173	Total 3018	C 975	H 1534	N 224	O 277	S 8	0	19	0

- Molecule 2 is 9-DEAZAGUANINE (three-letter code: 9DG) (formula: unknown).

Mol	Chain	Residues	Atoms					ZeroOcc	AltConf
2	C	1	Total 17	C 6	H 6	N 4	O 1	0	0
2	C	1	Total 17	C 6	H 6	N 4	O 1	0	0

- Molecule 3 is ALPHA-PHOSPHORIBOSYLPYROPHOSPHORIC ACID (three-letter code: PRP) (formula: unknown).

Mol	Chain	Residues	Atoms					ZeroOcc	AltConf
3	D	1	Total 30	C 5	H 8	O 14	P 3	0	0
3	D	1	Total 30	C 5	H 8	O 14	P 3	0	0

- Molecule 4 is MAGNESIUM ION (three-letter code: MG) (formula: unknown).

Mol	Chain	Residues	Atoms		ZeroOcc	AltConf
4	E	1	Total 1	Mg 1	0	0
4	E	1	Total 1	Mg 1	0	0
4	E	1	Total 1	Mg 1	0	0

Continued on next page...

PRELIMINARY VALIDATION REPORT

Continued from previous page...

Mol	Chain	Residues	Atoms		ZeroOcc	AltConf
			Total	Mg		
4	E	1	1	1	0	0

- Molecule 5 is DI(HYDROXYETHYL)ETHER (three-letter code: PEG) (formula: unknown).

Mol	Chain	Residues	Atoms				ZeroOcc	AltConf
			Total	C	H	O		
5	G	1	17	4	10	3	0	0
5	G	1	17	4	10	3	0	0
5	G	1	17	4	10	3	0	0
5	G	1	17	4	10	3	0	0
5	G	1	17	4	10	3	0	0

- Molecule 6 is ACETATE ION (three-letter code: ACT) (formula: unknown).

Mol	Chain	Residues	Atoms				ZeroOcc	AltConf
			Total	C	H	O		
6	H	1	7	2	3	2	0	0
6	H	1	7	2	3	2	0	0
6	H	1	7	2	3	2	0	0

- Molecule 7 is 1,2-ETHANEDIOL (three-letter code: EDO) (formula: unknown).

Mol	Chain	Residues	Atoms				ZeroOcc	AltConf
			Total	C	H	O		
7	I	1	10	2	6	2	0	0
7	I	1	10	2	6	2	0	0
7	I	1	10	2	6	2	0	0
7	I	1	10	2	6	2	0	0
7	I	1	10	2	6	2	0	0
7	I	1	10	2	6	2	0	0

Continued on next page...

PRELIMINARY VALIDATION REPORT

Continued from previous page...

Mol	Chain	Residues	Atoms	ZeroOcc	AltConf
7	I	1	Total C H O<br>10 2 6 2	0	0
7	I	1	Total C H O<br>10 2 6 2	0	0
7	I	1	Total C H O<br>10 2 6 2	0	0
7	I	1	Total C H O<br>10 2 6 2	0	0
7	I	1	Total C H O<br>10 2 6 2	0	0

- Molecule 8 is water.

Mol	Chain	Residues	Atoms	ZeroOcc	AltConf
8	S	318	Total O<br>318 318	0	0

PRELIMINARY VALIDATION REPORT

3 Residue-property plots (i)

These plots are drawn for all protein, RNA and DNA chains in the entry. The first graphic for a chain summarises the proportions of the various outlier classes displayed in the second graphic. The second graphic shows the sequence view annotated by issues in geometry and electron density. Residues are color-coded according to the number of geometric quality criteria for which they contain at least one outlier: green = 0, yellow = 1, orange = 2 and red = 3 or more. A red dot above a residue indicates a poor fit to the electron density (RSRZ > 2). Stretches of 2 or more consecutive residues without any outlier are shown as a green connector. Residues present in the sample, but not in the model, are shown in grey.

- Molecule 1:

Chain A:

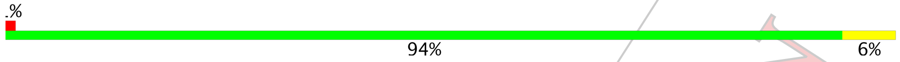

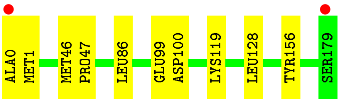

- Molecule 1:

Chain B:

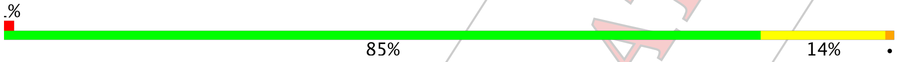

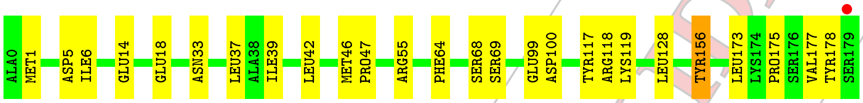

GLOBAL-STATISTICS INFOmissingINFO

PRELIMINARY VALIDATION REPORT

4 Model quality (i)

4.1 Standard geometry (i)

Bond lengths and bond angles in the following residue types are not validated in this section: MG, 9DG, EDO, PRP, ACT, PEG

The Z score for a bond length (or angle) is the number of standard deviations the observed value is removed from the expected value. A bond length (or angle) with $|Z| > 5$ is considered an outlier worth inspection. RMSZ is the root-mean-square of all Z scores of the bond lengths (or angles).

Mol	Chain	Bond lengths		Bond angles	
		RMSZ	$\#\|Z\| > 5$	RMSZ	$\#\|Z\| > 5$
1	A	0.48	0/1448	0.70	2/1954 (0.1%)
1	B	0.48	0/1542	0.71	2/2079 (0.1%)
All	All	0.48	0/2990	0.70	4/4033 (0.1%)

There are no bond length outliers.

All (4) bond angle outliers are listed below:

Mol	Chain	Res	Type	Atoms	Z	Observed(^{o})	Ideal(^{o})
1	A	100	ASP	CB-CG-OD2	-5.75	113.13	118.30
1	B	100	ASP	CB-CG-OD2	-5.54	113.32	118.30
1	B	100	ASP	CB-CG-OD1	5.41	123.17	118.30
1	A	100	ASP	CB-CG-OD1	5.04	122.84	118.30

There are no chirality outliers.

There are no planarity outliers.

4.2 Too-close contacts (i)

In the following table, the Non-H and H(model) columns list the number of non-hydrogen atoms and hydrogen atoms in the chain respectively. The H(added) column lists the number of hydrogen atoms added and optimized by MolProbity. The Clashes column lists the number of clashes within the asymmetric unit, whereas Symm-Clashes lists symmetry related clashes.

Mol	Chain	Non-H	H(model)	H(added)	Clashes	Symm-Clashes
1	A	1406	1431	1482	10	0
1	B	1484	1534	1588	19	0
2	C	22	12	12	0	0
3	D	44	16	12	0	0
4	E	4	0	0	0	0

Continued on next page...

PRELIMINARY VALIDATION REPORT

Continued from previous page...

Mol	Chain	Non-H	H(model)	H(added)	Clashes	Symm-Clashes
5	G	35	50	50	1	0
6	H	12	9	9	0	0
7	I	44	66	66	1	0
8	S	318	0	0	6	0
All	All	3369	3118	3219	26	0

The all-atom clashscore is defined as the number of clashes found per 1000 atoms (including hydrogen atoms). The all-atom clashscore for this structure is 4.

All (26) close contacts within the same asymmetric unit are listed below, sorted by their clash magnitude.

Atom-1	Atom-2	Interatomic distance (Å)	Clash overlap (Å)
1:A:46[A]:MET:SD	1:B:46[A]:MET:HE3	2.16	0.86
1:A:46[A]:MET:SD	1:B:46[A]:MET:CE	2.79	0.70
1:A:46[B]:MET:HG2	1:B:46[B]:MET:HG2	1.73	0.69
1:B:46[A]:MET:HE1	1:B:64:PHE:CE1	2.35	0.61
1:B:1:MET:HB2	8:S:336:HOH:O	2.06	0.54
1:B:37:LEU:HG	1:B:39[B]:ILE:HD11	1.90	0.53
1:B:175[A]:PRO:HA	1:B:178[A]:TYR:CE2	2.45	0.51
1:B:128:LEU:HD22	1:B:156[B]:TYR:HD2	1.78	0.48
1:B:175[A]:PRO:HA	1:B:178[A]:TYR:CD2	2.48	0.48
1:B:6:ILE:HD13	1:B:173[A]:LEU:HD23	1.96	0.48
1:A:0:ALA:HA	8:S:218:HOH:O	2.13	0.48
1:A:86:LEU:O	5:G:3:PEG:H31	2.13	0.47
1:B:117:TYR:O	1:B:119[A]:LYS:HE2	2.14	0.47
1:A:128:LEU:HD22	1:A:156[A]:TYR:HD2	1.80	0.47
1:B:33:ASN:ND2	8:S:180:HOH:O	2.40	0.46
1:A:1:MET:HB2	8:S:335:HOH:O	2.15	0.45
1:A:119:LYS:HE3	8:S:141:HOH:O	2.17	0.45
1:B:68:SER:O	1:B:69:SER:CB	2.65	0.45
1:A:46[B]:MET:HB3	1:A:47:PRO:HD3	2.00	0.44
1:B:5:ASP:HB3	1:B:177[B]:VAL:HG21	2.00	0.44
1:B:46[B]:MET:HB3	1:B:47:PRO:HD3	2.00	0.43
1:B:55:ARG:HE	7:I:6:EDO:C1	2.33	0.42
1:B:42:LEU:HD23	1:B:46[A]:MET:SD	2.60	0.42
1:A:1:MET:HG3	8:S:335:HOH:O	2.19	0.41
1:B:118:ARG:O	1:B:119[B]:LYS:HB2	2.21	0.40
1:B:14:GLU:O	1:B:18[A]:GLU:HG3	2.20	0.40

There are no symmetry-related clashes.

PRELIMINARY VALIDATION REPORT

4.3 Torsion angles (i)

4.3.1 Protein backbone (i)

In the following table, the Percentiles column shows the percent Ramachandran outliers of the chain as a percentile score with respect to all X-ray entries followed by that with respect to entries of similar resolution.

The Analysed column shows the number of residues for which the backbone conformation was analysed, and the total number of residues.

Mol	Chain	Analysed	Favoured	Allowed	Outliers	Percentiles	
1	A	176/173 (102%)	174 (99%)	2 (1%)	0	100	100
1	B	187/173 (108%)	183 (98%)	4 (2%)	0	100	100
All	All	363/346 (105%)	357 (98%)	6 (2%)	0	100	100

There are no Ramachandran outliers to report.

4.3.2 Protein sidechains (i)

In the following table, the Percentiles column shows the percent sidechain outliers of the chain as a percentile score with respect to all X-ray entries followed by that with respect to entries of similar resolution.

The Analysed column shows the number of residues for which the sidechain conformation was analysed, and the total number of residues.

Mol	Chain	Analysed	Rotameric	Outliers	Percentiles	
1	A	160/153 (105%)	159 (99%)	1 (1%)	89	79
1	B	172/153 (112%)	169 (98%)	3 (2%)	66	40
All	All	332/306 (108%)	328 (99%)	4 (1%)	80	55

All (4) residues with a non-rotameric sidechain are listed below:

Mol	Chain	Res	Type
1	A	99	GLU
1	B	99	GLU
1	B	156[A]	TYR
1	B	156[B]	TYR

Some sidechains can be flipped to improve hydrogen bonding and reduce clashes. There are no such sidechains identified.

PRELIMINARY VALIDATION REPORT

4.3.3 RNA (i)

There are no RNA molecules in this entry.

4.4 Non-standard residues in protein, DNA, RNA chains (i)

There are no non-standard protein/DNA/RNA residues in this entry.

4.5 Carbohydrates (i)

There are no carbohydrates in this entry.

4.6 Ligand geometry (i)

Of 27 ligands modelled in this entry, 4 are modelled with single atom - leaving 23 for Mogul analysis.

In the following table, the Counts columns list the number of bonds (or angles) for which Mogul statistics could be retrieved, the number of bonds (or angles) that are observed in the model and the number of bonds (or angles) that are defined in the chemical component dictionary. The Link column lists molecule types, if any, to which the group is linked. The Z score for a bond length (or angle) is the number of standard deviations the observed value is removed from the expected value. A bond length (or angle) with $|Z| > 2$ is considered an outlier worth inspection. RMSZ is the root-mean-square of all Z scores of the bond lengths (or angles).

Mol	Type	Chain	Res	Link	Bond lengths			Bond angles		
					Counts	RMSZ	$\#\|Z\| > 2$	Counts	RMSZ	$\#\|Z\| > 2$
2	9DG	C	1	-	12,?,?	2.83	7 (58%)	16,?,?	2.10	6 (37%)
2	9DG	C	2	-	12,?,?	2.80	7 (58%)	16,?,?	2.07	5 (31%)
3	PRP	D	403	4	20,?,?	4.14	8 (40%)	30,?,?	1.40	5 (16%)
3	PRP	D	404	4	20,?,?	4.04	7 (35%)	30,?,?	1.42	5 (16%)
5	PEG	G	1	-	6,?,?	0.52	0	5,?,?	0.32	0
5	PEG	G	2	-	6,?,?	0.52	0	5,?,?	0.34	0
5	PEG	G	3	-	6,?,?	0.46	0	5,?,?	0.27	0
5	PEG	G	4	-	6,?,?	0.49	0	5,?,?	0.46	0
5	PEG	G	5	-	6,?,?	0.52	0	5,?,?	0.24	0
6	ACT	H	1	-	3,?,?	1.21	0	3,?,?	1.38	0
6	ACT	H	2	-	3,?,?	1.33	0	3,?,?	1.44	0
6	ACT	H	3	-	3,?,?	1.32	0	3,?,?	1.52	0
7	EDO	I	1	-	3,?,?	0.48	0	2,?,?	0.15	0
7	EDO	I	10	-	3,?,?	0.45	0	2,?,?	0.49	0
7	EDO	I	11	-	3,?,?	0.42	0	2,?,?	0.30	0
7	EDO	I	2	-	3,?,?	0.45	0	2,?,?	0.44	0

PRELIMINARY VALIDATION REPORT

Mol	Type	Chain	Res	Link	Bond lengths			Bond angles		
					Counts	RMSZ	#|Z| > 2	Counts	RMSZ	#|Z| > 2
7	EDO	I	3	-	3,?,?	0.43	0	2,?,?	0.33	0
7	EDO	I	4	-	3,?,?	0.44	0	2,?,?	0.59	0
7	EDO	I	5	-	3,?,?	0.51	0	2,?,?	0.41	0
7	EDO	I	6	-	3,?,?	0.49	0	2,?,?	0.28	0
7	EDO	I	7	-	3,?,?	0.49	0	2,?,?	0.30	0
7	EDO	I	8	-	3,?,?	0.54	0	2,?,?	0.24	0
7	EDO	I	9	-	3,?,?	0.57	0	2,?,?	0.13	0

In the following table, the Chirals column lists the number of chiral outliers, the number of chiral centers analysed, the number of these observed in the model and the number defined in the chemical component dictionary. Similar counts are reported in the Torsion and Rings columns. '-' means no outliers of that kind were identified.

Mol	Type	Chain	Res	Link	Chirals	Torsions	Rings
2	9DG	C	1	-	-	0/0/?/?	0/2/?/?
2	9DG	C	2	-	-	0/0/?/?	0/2/?/?
3	PRP	D	403	4	-	0/16/?/?	0/1/?/?
3	PRP	D	404	4	-	0/16/?/?	0/1/?/?
5	PEG	G	1	-	-	0/4/?/?	0/0/?/?
5	PEG	G	2	-	-	0/4/?/?	0/0/?/?
5	PEG	G	3	-	-	0/4/?/?	0/0/?/?
5	PEG	G	4	-	-	0/4/?/?	0/0/?/?
5	PEG	G	5	-	-	0/4/?/?	0/0/?/?
6	ACT	H	1	-	-	0/0/?/?	0/0/?/?
6	ACT	H	2	-	-	0/0/?/?	0/0/?/?
6	ACT	H	3	-	-	0/0/?/?	0/0/?/?
7	EDO	I	1	-	-	0/1/?/?	0/0/?/?
7	EDO	I	10	-	-	0/1/?/?	0/0/?/?
7	EDO	I	11	-	-	0/1/?/?	0/0/?/?
7	EDO	I	2	-	-	0/1/?/?	0/0/?/?
7	EDO	I	3	-	-	0/1/?/?	0/0/?/?
7	EDO	I	4	-	-	0/1/?/?	0/0/?/?
7	EDO	I	5	-	-	0/1/?/?	0/0/?/?
7	EDO	I	6	-	-	0/1/?/?	0/0/?/?
7	EDO	I	7	-	-	0/1/?/?	0/0/?/?
7	EDO	I	8	-	-	0/1/?/?	0/0/?/?
7	EDO	I	9	-	-	0/1/?/?	0/0/?/?

All (29) bond length outliers are listed below:

Mol	Chain	Res	Type	Atoms	Z	Observed(Å)	Ideal(Å)
3	D	403	PRP	C1-C2	-12.25	1.37	1.52

Continued on next page...

PRELIMINARY VALIDATION REPORT

Continued from previous page...

Mol	Chain	Res	Type	Atoms	Z	Observed(Å)	Ideal(Å)
3	D	404	PRP	C1-C2	-11.90	1.37	1.52
2	C	1	9DG	C9-C4	-4.94	1.34	1.45
2	C	2	9DG	C9-C4	-4.86	1.34	1.45
3	D	403	PRP	O4-C4	-4.47	1.34	1.45
3	D	404	PRP	O4-C4	-4.18	1.35	1.45
3	D	404	PRP	O3-C3	-2.03	1.38	1.43
3	D	404	PRP	C3-C4	2.03	1.58	1.53
3	D	403	PRP	O2-C2	2.07	1.47	1.43
3	D	403	PRP	PB-O3A	2.26	1.63	1.60
3	D	403	PRP	C3-C4	2.33	1.59	1.53
3	D	403	PRP	P-O5	2.40	1.67	1.60
3	D	403	PRP	PA-O1	2.41	1.66	1.60
2	C	2	9DG	C2-N2	2.58	1.40	1.34
3	D	404	PRP	PA-O1	2.60	1.67	1.60
3	D	404	PRP	P-O5	2.62	1.68	1.60
2	C	1	9DG	C2-N2	2.69	1.40	1.34
2	C	2	9DG	C6-N1	2.92	1.44	1.38
2	C	1	9DG	C6-N1	2.98	1.44	1.38
2	C	2	9DG	C2-N1	2.98	1.45	1.37
2	C	1	9DG	C2-N1	3.02	1.45	1.37
2	C	1	9DG	C2-N3	3.34	1.41	1.33
2	C	2	9DG	C2-N3	3.38	1.41	1.33
2	C	2	9DG	C4-N3	3.54	1.46	1.38
2	C	1	9DG	C4-N3	3.86	1.47	1.38
2	C	1	9DG	C5-C6	3.92	1.52	1.42
2	C	2	9DG	C5-C6	4.16	1.52	1.42
3	D	404	PRP	O4-C1	11.59	1.62	1.41
3	D	403	PRP	O4-C1	11.76	1.62	1.41

All (21) bond angle outliers are listed below:

Mol	Chain	Res	Type	Atoms	Z	Observed(^{o})	Ideal(^{o})
2	C	2	9DG	C5-C4-N3	-5.56	120.10	124.71
2	C	1	9DG	C5-C4-N3	-5.54	120.11	124.71
2	C	2	9DG	C2-N1-C6	-3.08	119.30	125.08
2	C	1	9DG	C2-N1-C6	-3.01	119.42	125.08
3	D	404	PRP	O4-C1-C2	-2.91	101.15	104.97
3	D	403	PRP	O4-C1-C2	-2.58	101.59	104.97
2	C	2	9DG	O6-C6-N1	-2.57	115.16	120.13
3	D	404	PRP	O2-C2-C3	-2.40	104.14	111.83
3	D	403	PRP	O2P-P-O5	-2.26	100.72	106.73
2	C	1	9DG	O6-C6-N1	-2.11	116.04	120.13

Continued on next page...

PRELIMINARY VALIDATION REPORT

Continued from previous page...

Mol	Chain	Res	Type	Atoms	Z	Observed(°)	Ideal(°)
2	C	2	9DG	C4-N3-C2	2.03	119.74	116.55
2	C	1	9DG	C5-C6-N1	2.08	118.53	112.31
2	C	1	9DG	C4-N3-C2	2.18	119.97	116.55
2	C	2	9DG	C9-C4-N3	2.34	129.92	122.46
2	C	1	9DG	C9-C4-N3	2.39	130.09	122.46
3	D	404	PRP	O1-C1-C2	2.40	110.86	106.70
3	D	403	PRP	O1-C1-C2	2.42	110.89	106.70
3	D	403	PRP	O5-P-O3P	2.65	113.92	106.47
3	D	404	PRP	O5-P-O3P	2.74	114.15	106.47
3	D	404	PRP	C1-C2-C3	3.08	106.25	102.29
3	D	403	PRP	C1-C2-C3	3.32	106.56	102.29

There are no chirality outliers.

There are no torsion outliers.

There are no ring outliers.

2 monomers are involved in 2 short contacts:

Mol	Chain	Res	Type	Clashes	Symm-Clashes
5	G	3	PEG	1	0
7	I	6	EDO	1	0

4.7 Other polymers (i)

There are no such residues in this entry.

4.8 Polymer linkage issues (i)

The following chains have linkage breaks:

Mol	Chain	Number of breaks
1	B	1
1	A	1

All chain breaks are listed below:

Model	Chain	Residue-1	Atom-1	Residue-2	Atom-2	Distance (Å)
1	B	69:SER	C	77:THR	N	7.64
1	A	69:SER	C	77:THR	N	6.26

PRELIMINARY VALIDATION REPORT

5 Fit of model and data (i)

5.1 Protein, DNA and RNA chains (i)

In the following table, the column labelled '#RSRZ> 2' contains the number (and percentage) of RSRZ outliers, followed by percent RSRZ outliers for the chain as percentile scores relative to all X-ray entries and entries of similar resolution. The OWAB column contains the minimum, median, 95th percentile and maximum values of the occupancy-weighted average B-factor per residue. The column labelled 'Q< 0.9' lists the number of (and percentage) of residues with an average occupancy less than 0.9.

Mol	Chain	Analysed	<RSRZ>	#RSRZ>2			OWAB(Å^2)	Q<0.9
1	A	173/173 (100%)	-0.60	2 (1%)	79	80	14, 21, 47, 61	0
1	B	173/173 (100%)	-0.58	1 (0%)	89	89	14, 21, 47, 61	0
All	All	346/346 (100%)	-0.59	3 (0%)	84	85	14, 21, 47, 61	0

All (3) RSRZ outliers are listed below:

Mol	Chain	Res	Type	RSRZ
1	B	179[A]	SER	2.9
1	A	179	SER	2.7
1	A	0	ALA	2.1

5.2 Non-standard residues in protein, DNA, RNA chains (i)

There are no non-standard protein/DNA/RNA residues in this entry.

5.3 Carbohydrates (i)

There are no carbohydrates in this entry.

5.4 Ligands (i)

In the following table, the Atoms column lists the number of modelled atoms in the group and the number defined in the chemical component dictionary. LLDF column lists the quality of electron density of the group with respect to its neighbouring residues in protein, DNA or RNA chains. The B-factors column lists the minimum, median, 95th percentile and maximum values of B factors of atoms in the group. The column labelled 'Q< 0.9' lists the number of atoms with occupancy less than 0.9.

PRELIMINARY VALIDATION REPORT

Mol	Type	Chain	Res	Atoms	RSCC	RSR	LLDF	B-factors($Å^2$)	Q<0.9
7	EDO	I	8	4/?	0.93	0.11	4.91	33,45,69,83	0
7	EDO	I	3	4/?	0.77	0.12	4.86	40,48,58,64	0
7	EDO	I	11	4/?	0.92	0.12	4.54	36,44,50,60	0
7	EDO	I	10	4/?	0.89	0.14	3.40	41,51,72,86	0
7	EDO	I	6	4/?	0.95	0.13	3.15	33,56,70,70	0
5	PEG	G	4	7/?	0.78	0.17	2.78	60,78,118,118	0
6	ACT	H	1	4/?	0.94	0.11	2.62	41,49,49,68	0
6	ACT	H	3	4/?	0.85	0.12	2.13	42,51,60,66	0
5	PEG	G	3	7/?	0.91	0.10	1.21	46,59,71,82	0
7	EDO	I	5	4/?	0.83	0.11	0.95	49,64,76,77	0
7	EDO	I	9	4/?	0.94	0.10	0.90	39,57,69,69	0
2	9DG	C	2	11/?	0.95	0.07	0.73	15,19,25,27	0
2	9DG	C	1	11/?	0.94	0.07	0.65	15,18,26,26	0
5	PEG	G	2	7/?	0.95	0.09	0.57	37,46,65,74	0
5	PEG	G	1	7/?	0.94	0.11	0.01	37,47,68,72	0
4	MG	E	3	1/?	0.98	0.06	-0.25	16,16,16,16	0
3	PRP	D	403	22/?	0.99	0.06	-0.36	14,17,22,23	0
3	PRP	D	404	22/?	0.99	0.06	-0.39	14,17,22,23	0
7	EDO	I	1	4/?	0.88	0.07	-0.80	42,51,60,72	0
6	ACT	H	2	4/?	0.92	0.07	-0.87	42,50,52,62	0
4	MG	E	4	1/?	0.99	0.04	-2.34	16,16,16,16	0
4	MG	E	2	1/?	0.98	0.04	-	14,14,14,14	0
4	MG	E	1	1/?	0.99	0.04	-	14,14,14,14	0
7	EDO	I	4	4/?	0.94	0.09	-	39,50,66,79	0
7	EDO	I	7	4/?	0.79	0.16	-	58,70,78,78	0
7	EDO	I	2	4/?	0.91	0.10	-	43,52,57,62	0
5	PEG	G	5	7/?	0.92	0.08	-	50,65,78,78	17

5.5 Other polymers (i)

There are no such residues in this entry.

PRELIMINARY VALIDATION REPORT
