# Supplementary material for: Evolution of (p)ppGpp-HPRT regulation through diversification of an allosteric oligomeric interaction
Source: eLife. 2019 Sep 25;8:e47534. doi: 10.7554/eLife.47534 (PMC6783271; doi:10.7554/eLife.47534)
Supplement: Table 2—source data 3. [file elife-47534-table2-data3.pdf]

Preliminary Full wwPDB X-ray Structure Validation Report (i)

Apr 17, 2018 – 06:39 PM EDT

Deposition ID : D_1000233140
PDB ID : *(not yet assigned)*

This is a Preliminary Full wwPDB X-ray Structure Validation Report.

This report is produced by the wwPDB Deposition System during initial deposition but before annotation of the structure.

We welcome your comments at *validation@mail.wwpdb.org*
A user guide is available at
https://www.wwpdb.org/validation/2017/XrayValidationReportHelp
with specific help available everywhere you see the (i) symbol.

PRELIMINARY VALIDATION REPORT

The following versions of software and data (see references (i)) were used in the production of this report:

MolProbity	:	4.02b-467
Mogul	:	1.7.3 (157068), CSD as539be (2018)
Xtriage (Phenix)	:	1.13
EDS	:	rb-20031021
Percentile statistics	:	20171227.v01 (using entries in the PDB archive December 27th 2017)
Refmac	:	5.8.0158
CCP4	:	7.0 (Gargrove)
Ideal geometry (proteins)	:	Engh & Huber (2001)
Ideal geometry (DNA, RNA)	:	Parkinson et al. (1996)
Validation Pipeline (wwPDB-VP)	:	rb-20031021

1 Overall quality at a glance (i)

The following experimental techniques were used to determine the structure:
X-RAY DIFFRACTION

The reported resolution of this entry is 2.10 Å.

Percentile scores (ranging between 0-100) for global validation metrics of the entry are shown in the following graphic. The table shows the number of entries on which the scores are based.

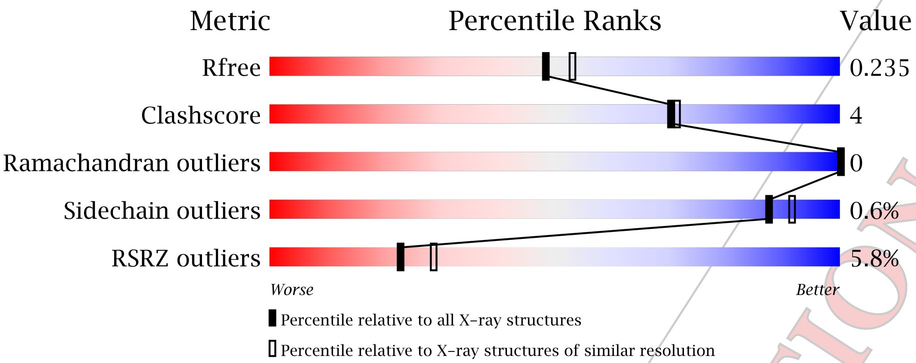

Metric	Whole archive (#Entries)	Similar resolution (#Entries, resolution range(Å))
R_{free}	111664	4608 (2.10-2.10)
Clashscore	122126	5109 (2.10-2.10)
Ramachandran outliers	120053	5059 (2.10-2.10)
Sidechain outliers	120020	5060 (2.10-2.10)
RSRZ outliers	108989	4497 (2.10-2.10)

The table below summarises the geometric issues observed across the polymeric chains and their fit to the electron density. The red, orange, yellow and green segments on the lower bar indicate the fraction of residues that contain outliers for >=3, 2, 1 and 0 types of geometric quality criteria. A grey segment represents the fraction of residues that are not modelled. The numeric value for each fraction is indicated below the corresponding segment, with a dot representing fractions <=5% The upper red bar (where present) indicates the fraction of residues that have poor fit to the electron density. The numeric value is given above the bar.

Mol	Chain	Length	Quality of chain
1	A	183	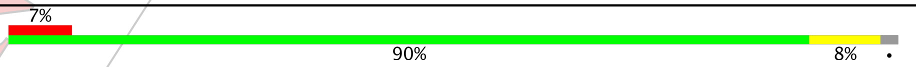
1	B	183	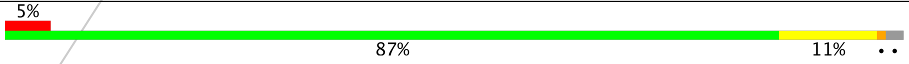

The following table lists non-polymeric compounds, carbohydrate monomers and non-standard residues in protein, DNA, RNA chains that are outliers for geometric or electron-density-fit criteria:

PRELIMINARY VALIDATION REPORT

Mol	Type	Chain	Res	Chirality	Geometry	Clashes	Electron density
4	PEG	E	8	-	-	-	X
4	PEG	E	9	-	-	-	X

PRELIMINARY VALIDATION REPORT

2 Entry composition (i)

There are 5 unique types of molecules in this entry. The entry contains 6086 atoms, of which 3010 are hydrogens and 0 are deuteriums.

In the tables below, the ZeroOcc column contains the number of atoms modelled with zero occupancy, the AltConf column contains the number of residues with at least one atom in alternate conformation and the Trace column contains the number of residues modelled with at most 2 atoms.

- Molecule 1 is a protein called hypoxanthine phosphoribosyltransferase (HPRT).

Mol	Chain	Residues	Atoms						ZeroOcc	AltConf	Trace
			Total	C	H	N	O	S			
1	A	180	2884	918	1464	221	274	7	0	1	0
1	B	180	2881	917	1464	220	273	7	0	1	0

- Molecule 2 is GUANOSINE-5',3'-TETRAPHOSPHATE (three-letter code: G4P) (formula: $C_{10}H_{17}N_5O_{17}P_4$).

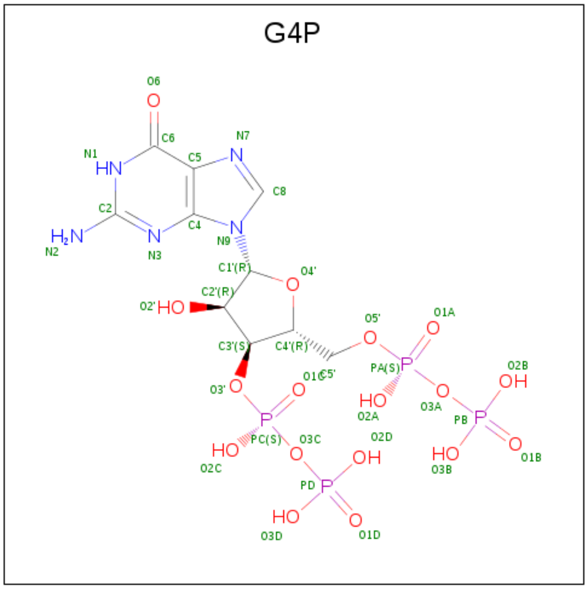

Mol	Chain	Residues	Atoms						ZeroOcc	AltConf
			Total	C	H	N	O	P		
2	C	1	47	10	11	5	17	4	0	0
2	C	1	47	10	11	5	17	4	0	0

- Molecule 3 is MAGNESIUM ION (three-letter code: MG) (formula: Mg).

PRELIMINARY VALIDATION REPORT

Mol	Chain	Residues	Atoms	ZeroOcc	AltConf
3	D	1	Total Mg<br>1 1	0	0
3	D	1	Total Mg<br>1 1	0	0

- Molecule 4 is DI(HYDROXYETHYL)ETHER (three-letter code: PEG) (formula: $C_4H_{10}O_3$).

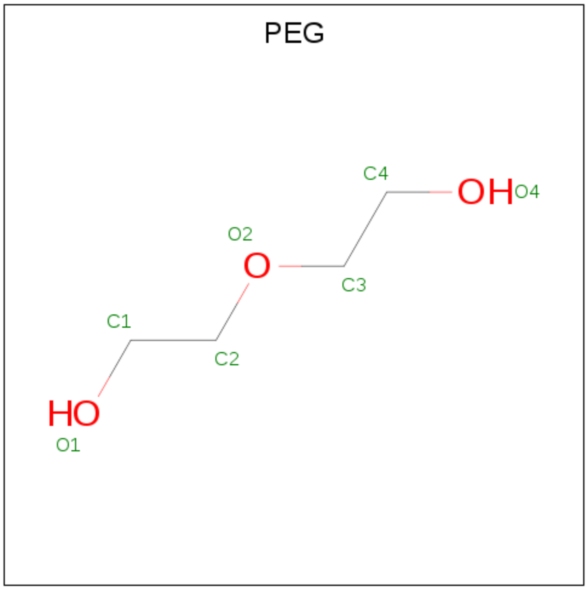

Mol	Chain	Residues	Atoms	ZeroOcc	AltConf
4	E	1	Total C H O<br>17 4 10 3	0	0
4	E	1	Total C H O<br>17 4 10 3	0	0
4	E	1	Total C H O<br>17 4 10 3	0	0
4	E	1	Total C H O<br>17 4 10 3	0	0
4	E	1	Total C H O<br>17 4 10 3	0	0
4	E	1	Total C H O<br>17 4 10 3	0	0

- Molecule 5 is water.

Mol	Chain	Residues	Atoms	ZeroOcc	AltConf
5	S	123	Total O<br>123 123	0	0

PRELIMINARY VALIDATION REPORT

3 Residue-property plots (i)

These plots are drawn for all protein, RNA and DNA chains in the entry. The first graphic for a chain summarises the proportions of the various outlier classes displayed in the second graphic. The second graphic shows the sequence view annotated by issues in geometryand electron density. Residues are color-coded according to the number of geometric quality criteria for which they contain at least one outlier: green = 0, yellow = 1, orange = 2 and red = 3 or more. A red dot above a residue indicates a poor fit to the electron density (RSRZ > 2). Stretches of 2 or more consecutive residues without any outlier are shown as a green connector. Residues present in the sample, but not in the model, are shown in grey.

- Molecule 1: hypoxanthine phosphoribosyltransferase (HPRT)

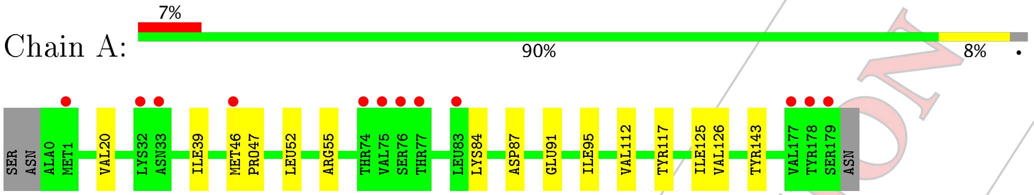

- Molecule 1: hypoxanthine phosphoribosyltransferase (HPRT)

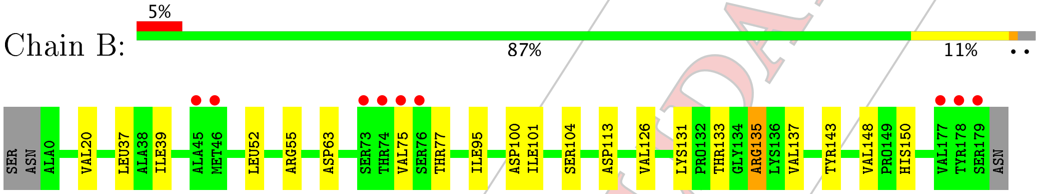

PRELIMINARY VALIDATION REPORT

4 Data and refinement statistics (i)

Property	Value	Source
Space group	P 31 2 1	Depositor
Cell constants<br>a, b, c, α, β, γ	82.61Å 82.61Å 174.92Å<br>90.00° 90.00° 120.00°	Depositor
Resolution (Å)	40.20 – 2.10<br>40.20 – 2.11	Depositor<br>EDS
% Data completeness<br>(in resolution range)	98.9 (40.20-2.10)<br>98.9 (40.20-2.11)	Depositor<br>EDS
R_{merge}	(Not available)	Depositor
R_{sym}	(Not available)	Depositor
$< I/\sigma(I) >$ [1]	2.03 (at 2.10Å)	Xtriage
Refinement program	PHENIX (1.13-3092)	Depositor
R, R_{free}	0.200 , 0.235<br>0.200 , 0.235	Depositor<br>DCC
R_{free} test set	1986 reflections (4.91%)	wwPDB-VP
Wilson B-factor (Å^2)	54.1	Xtriage
Anisotropy	0.102	Xtriage
Bulk solvent k_{sol}(e/Å^3), B_{sol}(Å^2)	0.39 , 73.4	EDS
L-test for twinning[2]	$< \|L\| >$ = 0.51, $< L^2 >$ = 0.34	Xtriage
Estimated twinning fraction	0.025 for -h,-k,l	Xtriage
F_o,F_c correlation	0.97	EDS
Total number of atoms	6086	wwPDB-VP
Average B, all atoms (Å^2)	83.0	wwPDB-VP

Xtriage's analysis on translational NCS is as follows: *The largest off-origin peak in the Patterson function is 3.21% of the height of the origin peak. No significant pseudotranslation is detected.*

[1] Intensities estimated from amplitudes.

[2] Theoretical values of $< |L| >$, $< L^2 >$ for acentric reflections are 0.5, 0.333 respectively for untwinned datasets, and 0.375, 0.2 for perfectly twinned datasets.

PRELIMINARY VALIDATION REPORT

5 Model quality (i)

5.1 Standard geometry (i)

Bond lengths and bond angles in the following residue types are not validated in this section: G4P, MG, PEG

The Z score for a bond length (or angle) is the number of standard deviations the observed value is removed from the expected value. A bond length (or angle) with $|Z| > 5$ is considered an outlier worth inspection. RMSZ is the root-mean-square of all Z scores of the bond lengths (or angles).

Mol	Chain	Bond lengths		Bond angles	
		RMSZ	$\#\|Z\| >5$	RMSZ	$\#\|Z\| >5$
1	A	0.26	0/1442	0.46	0/1950
1	B	0.26	0/1442	0.47	0/1950
All	All	0.26	0/2884	0.47	0/3900

There are no bond length outliers.

There are no bond angle outliers.

There are no chirality outliers.

There are no planarity outliers.

5.2 Too-close contacts (i)

In the following table, the Non-H and H(model) columns list the number of non-hydrogen atoms and hydrogen atoms in the chain respectively. The H(added) column lists the number of hydrogen atoms added and optimized by MolProbity. The Clashes column lists the number of clashes within the asymmetric unit, whereas Symm-Clashes lists symmetry related clashes.

Mol	Chain	Non-H	H(model)	H(added)	Clashes	Symm-Clashes
1	A	1420	1464	1464	11	0
1	B	1417	1464	1464	16	0
2	C	72	22	22	1	0
3	D	2	0	0	0	0
4	E	42	60	60	0	0
5	S	123	0	0	1	0
All	All	3076	3010	3010	23	0

The all-atom clashscore is defined as the number of clashes found per 1000 atoms (including hydrogen atoms). The all-atom clashscore for this structure is 4.

All (23) close contacts within the same asymmetric unit are listed below, sorted by their clash

PRELIMINARY VALIDATION REPORT

magnitude.

Atom-1	Atom-2	Interatomic distance (Å)	Clash overlap (Å)
1:A:91:GLU:OE1	1:B:77:THR:HG21	1.92	0.70
1:B:131:LYS:HG2	1:B:148:VAL:HG23	1.75	0.66
1:A:87:ASP:OD1	5:S:106:HOH:O	2.13	0.65
1:B:104:SER:N	2:C:2:G4P:O1B	2.38	0.56
1:B:133:THR:HG21	1:B:150:HIS:HB2	1.91	0.52
1:A:91:GLU:CD	1:B:77:THR:HG21	2.29	0.52
1:B:101:ILE:HD11	1:B:131:LYS:HE2	1.91	0.50
1:A:39:ILE:HD12	1:A:95:ILE:HG23	1.93	0.50
1:B:20:VAL:HG12	1:B:55:ARG:HG3	1.94	0.49
1:B:20:VAL:HG13	1:B:52:LEU:HA	1.95	0.48
1:A:91:GLU:OE2	1:B:77:THR:HG21	2.13	0.48
1:A:20:VAL:HG12	1:A:55:ARG:HG3	1.97	0.47
1:A:126:VAL:HG23	1:A:143:TYR:HB2	1.99	0.45
1:B:75:VAL:O	1:B:75:VAL:HG12	2.16	0.44
1:B:126:VAL:HG23	1:B:143:TYR:HB2	1.99	0.44
1:A:20:VAL:HG13	1:A:52:LEU:HA	2.00	0.44
1:B:39:ILE:HD12	1:B:95:ILE:HG23	1.98	0.44
1:A:46[A]:MET:HB3	1:A:47:PRO:HD3	1.99	0.44
1:B:100:ASP:OD1	1:B:100:ASP:N	2.52	0.43
1:B:135:ARG:HG3	1:B:137:VAL:O	2.19	0.42
1:A:112:VAL:HG21	1:A:125:ILE:HD11	2.00	0.42
1:A:117:TYR:OH	1:B:113:ASP:HB2	2.20	0.41
1:B:37:LEU:HD11	1:B:63:ASP:HB2	2.03	0.41

There are no symmetry-related clashes.

5.3 Torsion angles (i)

5.3.1 Protein backbone (i)

In the following table, the Percentiles column shows the percent Ramachandran outliers of the chain as a percentile score with respect to all X-ray entries followed by that with respect to entries of similar resolution.

The Analysed column shows the number of residues for which the backbone conformation was analysed, and the total number of residues.

Mol	Chain	Analysed	Favoured	Allowed	Outliers	Percentiles	
1	A	179/183 (98%)	169 (94%)	10 (6%)	0	100	100
1	B	179/183 (98%)	173 (97%)	6 (3%)	0	100	100

Continued on next page...

PRELIMINARY VALIDATION REPORT

Continued from previous page...

Mol	Chain	Analysed	Favoured	Allowed	Outliers	Percentiles	
All	All	358/366 (98%)	342 (96%)	16 (4%)	0	100	100

There are no Ramachandran outliers to report.

5.3.2 Protein sidechains (i)

In the following table, the Percentiles column shows the percent sidechain outliers of the chain as a percentile score with respect to all X-ray entries followed by that with respect to entries of similar resolution.

The Analysed column shows the number of residues for which the sidechain conformation was analysed, and the total number of residues.

Mol	Chain	Analysed	Rotameric	Outliers	Percentiles	
1	A	159/162 (98%)	158 (99%)	1 (1%)	87	91
1	B	159/162 (98%)	158 (99%)	1 (1%)	87	91
All	All	318/324 (98%)	316 (99%)	2 (1%)	87	91

All (2) residues with a non-rotameric sidechain are listed below:

Mol	Chain	Res	Type
1	A	84	LYS
1	B	135	ARG

Some sidechains can be flipped to improve hydrogen bonding and reduce clashes. There are no such sidechains identified.

5.3.3 RNA (i)

There are no RNA molecules in this entry.

5.4 Non-standard residues in protein, DNA, RNA chains (i)

There are no non-standard protein/DNA/RNA residues in this entry.

5.5 Carbohydrates (i)

There are no carbohydrates in this entry.

PRELIMINARY VALIDATION REPORT

5.6 Ligand geometry (i)

Of 10 ligands modelled in this entry, 2 are monoatomic - leaving 8 for Mogul analysis.

In the following table, the Counts columns list the number of bonds (or angles) for which Mogul statistics could be retrieved, the number of bonds (or angles) that are observed in the model and the number of bonds (or angles) that are defined in the Chemical Component Dictionary. The Link column lists molecule types, if any, to which the group is linked. The Z score for a bond length (or angle) is the number of standard deviations the observed value is removed from the expected value. A bond length (or angle) with $|Z| > 2$ is considered an outlier worth inspection. RMSZ is the root-mean-square of all Z scores of the bond lengths (or angles).

Mol	Type	Chain	Res	Link	Bond lengths			Bond angles		
					Counts	RMSZ	$\#\|Z\| > 2$	Counts	RMSZ	$\#\|Z\| > 2$
2	G4P	C	1	3	32,38,38	1.31	3 (9%)	35,61,61	1.74	7 (20%)
2	G4P	C	2	3	32,38,38	1.28	3 (9%)	35,61,61	1.78	7 (20%)
4	PEG	E	1	-	6,6,6	0.50	0	5,5,5	0.33	0
4	PEG	E	2	-	6,6,6	0.49	0	5,5,5	0.50	0
4	PEG	E	3	-	6,6,6	0.49	0	5,5,5	0.38	0
4	PEG	E	4	-	6,6,6	0.50	0	5,5,5	0.36	0
4	PEG	E	8	-	6,6,6	0.50	0	5,5,5	0.32	0
4	PEG	E	9	-	6,6,6	0.49	0	5,5,5	0.36	0

In the following table, the Chirals column lists the number of chiral outliers, the number of chiral centers analysed, the number of these observed in the model and the number defined in the Chemical Component Dictionary. Similar counts are reported in the Torsion and Rings columns. '-' means no outliers of that kind were identified.

Mol	Type	Chain	Res	Link	Chirals	Torsions	Rings
2	G4P	C	1	3	-	1/23/43/43	0/3/3/3
2	G4P	C	2	3	-	1/23/43/43	0/3/3/3
4	PEG	E	1	-	-	0/4/4/4	0/0/0/0
4	PEG	E	2	-	-	0/4/4/4	0/0/0/0
4	PEG	E	3	-	-	0/4/4/4	0/0/0/0
4	PEG	E	4	-	-	0/4/4/4	0/0/0/0
4	PEG	E	8	-	-	0/4/4/4	0/0/0/0
4	PEG	E	9	-	-	0/4/4/4	0/0/0/0

All (6) bond length outliers are listed below:

Mol	Chain	Res	Type	Atoms	Z	Observed(Å)	Ideal(Å)
2	C	1	G4P	C2-N2	2.24	1.35	1.32
2	C	2	G4P	C2-N2	2.33	1.35	1.32
2	C	2	G4P	C5-C4	2.88	1.47	1.40
2	C	1	G4P	C5-C4	2.90	1.47	1.40

Continued on next page...

PRELIMINARY VALIDATION REPORT

Continued from previous page...

Mol	Chain	Res	Type	Atoms	Z	Observed(Å)	Ideal(Å)
2	C	2	G4P	C6-C5	4.42	1.48	1.41
2	C	1	G4P	C6-C5	4.55	1.48	1.41

All (14) bond angle outliers are listed below:

Mol	Chain	Res	Type	Atoms	Z	Observed(^{o})	Ideal(^{o})
2	C	1	G4P	PA-O3A-PB	-4.25	118.33	132.63
2	C	2	G4P	PA-O3A-PB	-3.80	119.86	132.63
2	C	1	G4P	C4-C5-N7	-3.77	105.77	109.41
2	C	2	G4P	C4-C5-N7	-3.71	105.82	109.41
2	C	2	G4P	N2-C2-N3	-3.31	116.27	120.26
2	C	1	G4P	N2-C2-N3	-3.16	116.46	120.26
2	C	1	G4P	C1'-N9-C4	-2.61	122.13	126.64
2	C	2	G4P	C1'-N9-C4	-2.60	122.15	126.64
2	C	2	G4P	PC-O3C-PD	-2.56	124.02	132.63
2	C	1	G4P	PC-O3C-PD	-2.24	125.09	132.63
2	C	1	G4P	N2-C2-N1	2.11	120.05	117.84
2	C	2	G4P	N2-C2-N1	2.34	120.29	117.84
2	C	2	G4P	C2-N3-C4	4.19	120.05	115.16
2	C	1	G4P	C2-N3-C4	4.23	120.10	115.16

There are no chirality outliers.

All (2) torsion outliers are listed below:

Mol	Chain	Res	Type	Atoms
2	C	1	G4P	PC-O3'-C3'-C4'
2	C	2	G4P	PC-O3'-C3'-C4'

There are no ring outliers.

1 monomer is involved in 1 short contact:

Mol	Chain	Res	Type	Clashes	Symm-Clashes
2	C	2	G4P	1	0

5.7 Other polymers (i)

There are no such residues in this entry.

PRELIMINARY VALIDATION REPORT

5.8 Polymer linkage issues (i)

There are no chain breaks in this entry.

PRELIMINARY VALIDATION REPORT

6 Fit of model and data (i)

6.1 Protein, DNA and RNA chains (i)

In the following table, the column labelled '#RSRZ> 2' contains the number (and percentage) of RSRZ outliers, followed by percent RSRZ outliers for the chain as percentile scores relative to all X-ray entries and entries of similar resolution. The OWAB column contains the minimum, median, 95th percentile and maximum values of the occupancy-weighted average B-factor per residue. The column labelled 'Q< 0.9' lists the number of (and percentage) of residues with an average occupancy less than 0.9.

Mol	Chain	Analysed	<RSRZ>	#RSRZ>2			OWAB(Å^2)	Q<0.9
1	A	180/183 (98%)	0.22	12 (6%)	18	22	51, 67, 113, 171	0
1	B	180/183 (98%)	0.27	9 (5%)	29	35	50, 66, 120, 146	0
All	All	360/366 (98%)	0.24	21 (5%)	23	29	50, 67, 118, 171	0

All (21) RSRZ outliers are listed below:

Mol	Chain	Res	Type	RSRZ
1	A	177	VAL	5.7
1	B	177	VAL	4.4
1	B	75	VAL	4.3
1	A	77	THR	3.8
1	A	74	THR	3.7
1	A	179	SER	3.5
1	B	179	SER	3.5
1	A	75	VAL	3.4
1	A	76	SER	3.4
1	B	73	SER	3.1
1	B	74	THR	2.9
1	A	178	TYR	2.8
1	B	178	TYR	2.5
1	B	76	SER	2.4
1	A	83	LEU	2.4
1	B	46[A]	MET	2.3
1	A	32	LYS	2.2
1	A	46[A]	MET	2.1
1	A	33	ASN	2.0
1	B	45	ALA	2.0
1	A	1	MET	2.0

PRELIMINARY VALIDATION REPORT

6.2 Non-standard residues in protein, DNA, RNA chains (i)

There are no non-standard protein/DNA/RNA residues in this entry.

6.3 Carbohydrates (i)

There are no carbohydrates in this entry.

6.4 Ligands (i)

In the following table, the Atoms column lists the number of modelled atoms in the group and the number defined in the chemical component dictionary. The B-factors column lists the minimum, median, 95th percentile and maximum values of B factors of atoms in the group. The column labelled 'Q< 0.9' lists the number of atoms with occupancy less than 0.9.

Mol	Type	Chain	Res	Atoms	RSCC	RSR	B-factors($Å^2$)	Q<0.9
4	PEG	E	9	7/?	0.37	0.50	263,316,324,333	0
4	PEG	E	8	7/?	0.45	0.41	236,289,346,347	0
4	PEG	E	4	7/?	0.64	0.23	135,172,217,224	0
4	PEG	E	2	7/?	0.73	0.23	143,172,191,197	0
3	MG	D	4	1/?	0.75	0.08	86,86,86,86	0
4	PEG	E	3	7/?	0.83	0.12	78,98,124,128	0
2	G4P	C	2	36/?	0.88	0.23	56,121,323,342	0
4	PEG	E	1	7/?	0.90	0.16	118,142,165,165	0
3	MG	D	2	1/?	0.94	0.09	66,66,66,66	0
2	G4P	C	1	36/?	0.94	0.13	53,74,177,194	0

6.5 Other polymers (i)

There are no such residues in this entry.

PRELIMINARY VALIDATION REPORT
